# SMAD-Independent Down-Regulation of Caveolin-1 by TGF-β: Effects on Proliferation and Survival of Myofibroblasts

**DOI:** 10.1371/journal.pone.0116995

**Published:** 2015-02-06

**Authors:** Yan Y. Sanders, Zongbin Cui, Claude Jourdan Le Saux, Jeffrey C. Horowitz, Sunad Rangarajan, Ashish Kurundkar, Veena B. Antony, Victor J. Thannickal

**Affiliations:** 1 Division of Pulmonary, Allergy and Critical Care, Department of Medicine, University of Alabama at Birmingham, Birmingham, Alabama, 35294, United States of America; 2 Division of Pulmonary and Critical Care Medicine, Department of Internal Medicine, University of Michigan Medical School, Ann Arbor, Michigan, 48109, United States of America; 3 Division of Cardiology, Department of Medicine, University of Texas Health Science Center at San Antonio, San Antonio, Texas, 78229, United States of America; University of Kansas Medical Center, UNITED STATES

## Abstract

Transforming growth factor-β (TGF-β) mediates growth-inhibitory effects on most target cells via activation of the canonical SMAD signaling pathway. This growth-inhibitory activity may be coupled with cellular differentiation. Our studies demonstrate that TGF-β1 inhibits proliferation of primary, non-transformed human lung fibroblasts in association with the induction of myofibroblast differentiation. Differentiated myofibroblasts maintain the capacity to proliferate in response to exogenous mitogenic stimuli and are resistant to serum deprivation-induced apoptosis. These proliferative and anti-apoptotic properties of myofibroblasts are related, in part, to the down-regulation of caveolin-1 (Cav-1) by TGF-β1. Cav-1 down-regulation is mediated by early activation of p38 MAPK and does not require SMAD signaling. In contrast, myofibroblast differentiation is dependent on activation of the SMAD pathway, but not on p38 MAPK. Thus, combinatorial signaling by TGF-β1 of myofibroblast differentiation and down-regulation of Cav-1 by SMAD and p38 MAPK pathways, respectively, confer proliferative and apoptosis-resistant properties to myofibroblasts. Selective targeting of this SMAD-independent, p38-MAPK/Cav-1-dependent pathway is likely to be effective in the treatment of pathological conditions characterized by TGF-β signaling and myofibroblast activation.

## INTRODUCTION

Transforming growth factor-β1 (TGF-β1) regulates cell growth, differentiation and apoptosis in a cell- and context-specific manner; thus, both tumor-promoter and tumor-suppressive actions have been described [1,2]. TGF-β1 mediates cytostatic effects on most target cells, including B and T lymphocytes [3,4], epithelial cells [5] and endothelial cells [6,7]. In contrast, several studies have demonstrated the ability of TGF-β1 to promote mesenchymal cell proliferation, an effect that appears to be mediated primarily by indirect mechanisms involving the autocrine production of mitogenic growth factors [8–10] and/or their receptor(s) up-regulation [11,12]. Furthermore, over-expression of TGF-β1 in rat lung results in the emergence and proliferation of myofibroblasts in association with prolonged severe fibrosis [13]. Similarly, direct transfer of TGF-β1 gene into arteries stimulates fibrocellular hyperplasia [14]. Thus, understanding cellular/molecular mechanisms by which TGF-β1 promotes growth of mesenchymal cells, in particular myofibroblasts, is likely to be important in various pathological conditions characterized by myofibroblasts accumulation and activation [15,16].

Caveolin proteins are the principal components of caveolae, morphologically distinct plasma membrane invaginations present on many cell types, that regulates a number of cellular physiological functions [17]. Caveolin-1 (Cav-1) was identified as the original member of the caveolin gene family and is expressed primarily in non-muscle cells. Overexpression of Cav-1 in cells lacking endogenous caveolae results in the *de novo* formation of caveolae [18,19]; while targeted down-regulation of Cav-1 in cells containing caveolae results in loss of caveolae [20,21]. Cav-1 gene is primarily recognized as a tumor-suppressor [22,23], although tumor-promoter activities have been described in some contexts [24,25]. The phenotype of Cav-1 knock-out mice has recently been described and is most remarkable for distinct pulmonary defects characterized by endothelial cell hyperproliferation and fibrosis [26]. The potential roles of fibroblasts/myofibroblasts, the major extracellular matrix (ECM)-producing cells in mammals, in the context of Cav-1 deficiency, is less clear.

We have previously shown that TGF-β1 is a potent inducer of myofibroblast differentiation by mechanisms involving cell adhesion and activation of focal adhesion kinase (FAK) [27]. TGF-β1 also promotes an apoptosis-resistant phenotype by the p38 MAPK-dependent autocrine production of soluble growth factor(s) [28]. Furthermore, exogenous receptor tyrosine kinases (RTKs)-activating fibroblast growth factors mediate enhanced mitogenic responses in TGF-β1-differentiated myofibroblasts [12]. Interestingly, the apoptotic resistant phenotype of fibroblasts in idiopathic pulmonary fibrosis (IPF) also results from the down-regulation of Cav-1 via a PTEN/Akt-dependent pathway [29]. Cav-1 is typically expressed at high levels in terminally differentiated or quiescent cells; however, the regulation of Cav-1 during the induction of myofibroblast differentiation is not well defined. Recently it has been shown that TGF-β1 can induce miRNA-199a, which controls the down-regulation of Cav1 in TGF-β1 treated fibroblasts [30]. In this study, we examined the regulation of Cav-1 expression in non-transformed human lung fibroblasts that undergo myofibroblast differentiation in response to TGF-β1 stimulation. We describe, for the first time, a novel action of TGF-β1 to down-regulate Cav-1 expression via SMAD-*in*dependent and p38 MAPK-dependent mechanisms; this occurs concomitantly with the induction of myofibroblast differentiation via canonical SMAD signaling. Furthermore, we explore potential role(s) of Cav-1 in the regulation of myofibroblast proliferation and apoptosis resistance.

## EXPERIMENTAL PROCEDURES

### Reagents

Porcine platelet-derived TGF-β1 was obtained from R&D Systems, Minneapolis, MN. Rabbit polyclonal antibody (N-20) against a peptide at the amino terminus of human Cav-1 was purchased from Santa Cruz Biotechnology, Santa Cruz, CA. Goat polyclonal antibody to total SMAD2 and mouse monoclonal antibody to cyclin D1 (A-12) were from Santa Cruz Biotechnology. Mouse monoclonal antibodies to α-smooth muscle actin (α-SMA), β-actin and β-tubulin were obtained from Sigma, St. Louis, MO. Mouse monoclonal antibody to single-stranded DNA (*ss*DNA) was from Chemicon International, Temecula, CA. Secondary horseradish peroxidase (HRP)-conjugated anti-rabbit or Alexa flour 594 goat conjugated anti-mouse antibodies were obtained from Pierce, Rockford, IL or Life Technologies, Grand Island, NY. SB203580 was purchased from Calbiochem, La Jolla, CA. PD98059 was from Cell Signaling Technology. SB431542 was purchased from TOCRIS Bioscience, Avonmouth, UK. The concentrations used in this study were determined by our previous published studies [31]. All other reagents were obtained from Sigma, unless otherwise stated.

### Cell culture

Studies were performed in non-transformed early passage of human fetal lung fibroblasts (IMR-90, Institute for Medical Research, Camden, NJ); these cells typically undergo cellular senescence after 40–50 replications, supporting their “normal”, non-transformed phenotype. IMR-90 fibroblasts were grown in DMEM (GIBCO, Grand Island, NY) supplemented with 10% fetal calf serum (FBS; Hyclone Laboratories, Logan, UT), 100 units/ml penicillin, 100 μg/ml streptomycin, and 1.25 μg/ml amphotericin B (GIBCO). Cells were plated on 35 mm cell culture dishes or 96-well plates and incubated at 37°C in 5% CO_2_, 95% air. Medium containing 10% serum was changed every other day.

### Generation of stably over-expressing Cav-1α, Cav-1 shRNA, SMAD2 shRNA and p38 MAPK mutant cell lines

A PCR-based strategy for site-specific mutagenesis was used to generate a construct pRC/CMV-Cav1 for overexpression of Cav-1α in IMR-90 cells. The codon of methionine at position 32 in human Cav-1 cDNA was changed to leucine (ATG to TTG) without affecting the function of Cav-1α and to eliminate transcription of Cav-1β [32]. PCR primers used for this mutation include Cav1α-M1, 5′-AACAAGGCCTTGGCAGACGAGCT-GAGCGAGAAG-3′ and Cav1α-M2, 5′-CGTCTGCCAAGGCCTTGTTGTTGGGCTTG-TAG-3′. The sequence near the start codon of Cav-1α was replaced with a sequence containing a standard Kozak (GCCGCCATGG, start codon is underlined) to increase expression of Cav-1. The Cav-1α cDNA was then subcloned into the *Hin*d III/*Xba* I site of an expression vector, pRC/CMV2 from Invitrogen. Primers used for PCR were: Cav1α-1, 5′-TTTTAAGCTTGCC-GCCATGGCTGGGGGCAAATACGTAG-3′ and Cav1α-2, 5′-GGGGTCTAGATTATAT-TTCTTTCTGCAAGTTGATG-3′.

A DNA-based *si*RNA expression vector with hygromycin resistance, p*Silencer* 2.1-U6 hygro from Ambion, was used to generate short hairpin RNA (*sh*RNA) for knockdown of Cav-1 and SMAD2 in human lung fibroblasts. The p*Silencer* 2.1-U6 hygro negative control plasmid supplied with the kit is a circular plasmid encoding a *sh*RNA whose sequence is not found in the mouse, human, or rat genome databases. To choose an effective target on Cav-1 and SMAD2 mRNA, three *sh*RNA expression constructs for distinctive targets were tested for each gene. Western blotting data showed that *sh*RNAs from three constructs including pSU6H-*sh*Cav1 and pSU6H-*sh*SMAD2 are able to knock down protein expression of Caveolin-1 and SMAD2 by 80–90% in IMR-90 cells. Oligonucleotides used for generating the construct pSU6H-*sh*Cav1 were: CA-2f: 5′-GATCCCACACCTCAACGATGACGTGTTCAAGAGACACGTCATCGTTGAGGGTTTTTTTGGAAA-3′, and CA-2r, 5′-AGCTTTTCCAAAAAAACACCTCAACGATGACGTGTCTCTTGAACACGTCATCGTTGAGGTGTGG-3′. Oligonucleotides used for generating the construct pSU6H-*sh*SMAD2 were: SMAD2-3f, 5′-GATCCCGTACACCAAATACGATAGATTCAAGAGATCTATCGTATTTGGTGTACTTTTTTGGAAA-3′ and SMAD2-3r, 5′-AGCTTTTCCAAAAAAGTACACCAAATACGATAGATCTCTTGAATCTATCGTATTTGGTGTACGG-3′. Sense and anti-sense strand sequences of Cav-1 and SMAD2 are underlined. Subcloning was performed according to established protocols from Ambion. Sequencing was performed by the University of Michigan Sequencing Core.

The mammalian expression plasmid, pcDNA-38KM encoding mutant p38 MAPK, was provided by Dr. Kun Liang Guan, Department of Biological Chemistry, University of Michigan, Ann Arbor. This p38 MAPK mutant protein is able to be phosphorylated, but is not catalytically active and unable to phosphorylate its downstream substrates such as ATF-2 [28]. Plasmid transfections of IMR-90 cells using the cationic lipid reagent, Lipofectamine (Invitrogen, Carlsbad, CA) were performed according to manufacturer’s instructions. Optimal ratio of DNA (μg) to Lipofectamine (μl) was determined to be ∼1:5 for IMR-90 cells. Cells were incubated with DNA-lipid complexes in serum-free Opti-MEM I medium (Invitrogen) for 4–5 h prior to introducing 10% FBS for 16–20 h. The next day, transfection medium was replaced by DMEM supplemented with 10% FBS and geneticin or hygromycin B (Invitrogen). Geneticin concentrations were 200–400 μg/ml for selection of stable transfectants and 150 μg/ml was the maintenance dose. Hygromycin B concentrations were 25–30 μg/ml for selection and 2 μg/ml was used as the maintenance dose. All cells were studied as pooled clones selected in geneticin or hygromycin and cells were then treated with/without TGF-β1 in the absence of selection reagents.

**Superarray cDNA microarray analyses and real-time quantitative RT-PCR:** RNA was isolated from cells using TRIzol reagent from Invitrogen according to the manufacturer’s protocol. Human extracellular matrix & adhesion molecules cDNA arrays from Superarray were used for the analyses of gene expression profiling in cells treated with/without TGF-β1 (2 ng/ml) for 24 h, according to manufacturer’s protocol (Superarray, Fredrick, MD). Cyclophilin A mRNA served as controls on these Superarray analyses.

Real-time quantitative RT-PCR was performed using iQ SYBR Green Supermix (BioRad, Palo Alto, CA) and using an iCylcer (BioRad) real-time detection system and normalized to 18S. Primers sequences are: Cav-1 F, 5′-GAGCTGAGCGAGAAGCAAGT-3′, Cav-1 R, 5′-TCCCTTCTGGTTCTGCAATC-3′. 18S F, 5′-GTCTGCCCTATCAACTTTCG-3′, 18S R, 5′-ATGTGGTAGCCGTTTCTCA-3’ as before [33]. Assays were performed in triplicate with 10ng of cDNA and 200 nM primers in a total reaction volume of 25 μl. Thermal cycling conditions were 95°C for 3 min, and 40 cycles of 95°C for 30 s, 60°C for 30 s.

### Apoptosis assays

Apoptosis was quantitated with the use of an ELISA-based assay for *ss*DNA (Apoptosis ELISA Kit, Chemicon International) according to the manufacturer’s instructions with minor modifications as previously described [28].

### Proliferation assay

Normal or stably transfected IMR-90 cells were seeded at 2 × 10^4^ cells per 35 mm dishes and grown in 10% FBS until 50–60% confluence. Cells were then serum-deprived for 24 h and then treated with/without TGF-β1 (2 ng/ml) for 48 h followed by stimulation with 10% FBS for 24 h. Cell counts were assessed both prior to and after serum stimulation with an automated series Z Coulter counter (Coulter Electronics, Hialeah, FL).

BrdU incorporation assays were performed using an assay kit purchased from Oncogene, San Diego, CA, according to manufacturer’s instructions. The “background” absorbance of cells receiving no BrdU label was subtracted and BrdU incorporation index was calculated by dividing the corrected absorbance by cell counts obtained prior to fixing the cells.

### Immunofluorescence staining and Western blotting

Immunofluoresence staining and Western immunoblotting procedures were performed as previously described [27].

### Statistical Analysis

Statistical analysis was performed using Student’s *t* test when comparing two groups and one-way analysis of variance with Bonferonni post-test when comparing three or more experimental conditions. This analysis was done using GraphPad Prism version 3.0 for Windows, GraphPad Software (San Diego, CA). Statistical significance was defined at *p* < 0.05. Densitometric analyses of Western blots were performed using the public domain NIH Image program available at http://rsb.info.nih.gov/nih-image.

## RESULTS

### TGF-β1 mediates growth inhibition and induction of myofibroblast differentiation in non-transformed human lung fibroblasts

**Figure 1:**
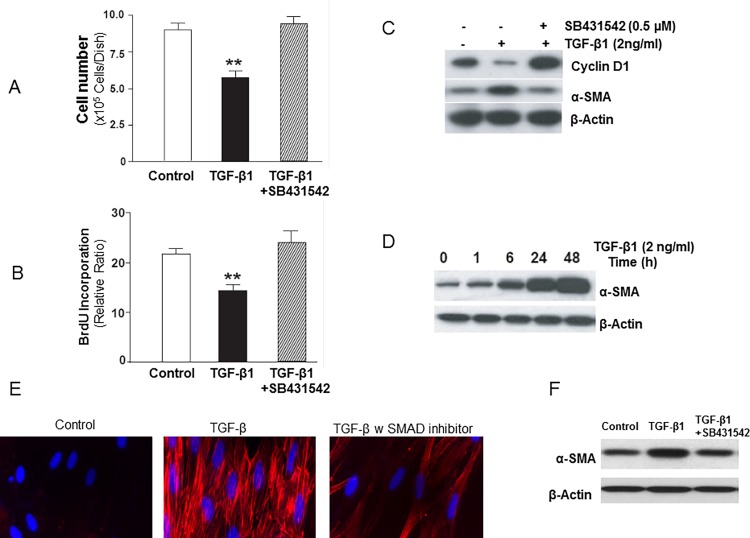
TGF-β1 induces growth suppression in association with induction of myofibroblast differentiation of human lung fibroblasts. ***A***, Early passage human lung fibroblasts (IMR-90), seeded at equal density on 35 mm dishes and grown to 50% confluence, were treated with or without TGF-β1 (2 ng/ml) in the presence/absence of the ALK5 inhibitor (SB431542, 0.5 μM) in 10% FBS for 48 h prior to assessments of cell numbers by Coulter counting (n = 6 per group). Results are averages of at least three independent experiments. Data are presented as mean±S.E.M. **indicates *p* < 0.01 vs. control cells or cells treated with TGF-β and SB431542. ***B***, IMR-90 cells as described in (A) were grown in 96-well plates and labeled with BrdU for 24 h prior to assays for BrdU incorporation (n = 6 per group). Results are averages of at least three independent experiments. Data are presented as mean±S.E.M. **indicates *p* < 0.01 vs. control cells or cells treated with TGF-β and SB431542. ***C***, IMR-90 cells grown in 10% FBS were stimulated with or without TGF-β1 (2 ng/ml) in the presence/absence of SB431542 (0.5 μM) for 48 h. Cell lysates were subjected to SDS-PAGE and immunoblotted with an antibody against Cyclin D1; the blot was then stripped and probed for α-smooth muscle actin (α-SMA) and β-actin. ***D***, IMR-90 cells as described in (A) were stimulated with or without TGF-β1 (2 ng/ml) for the indicated times. Western immunoblotting was performed with a monoclonal antibody against α-SMA; the blot was stripped and probed for β-actin. **E**, Immunofluorescent images of IMR-90 cells treated 24h with vehicle only control, TGF-β only (2ng/ml) and TGF-β (2ng/ml) with SMAD inhibitor (SB431542, 0.5 μM). Cells were stained with antibody specific for α-SMA (1:100) with Alexa flour 594 conjugated secondary antibody (red); and 4′,6-diamidino-2-phenylindole (DAPI, 300nM in PBS) staining was used to identify the nucleus (blue). Representative microscopy images are shown. Magnification of ×40, figures were obtained with a Zeiss Axiovert fluorescence microscope. **F**, α-SMA expression by western blot in IMR-90 cells treated 24h with vehicle only control, TGF-β only (2ng/ml) and TGF-β (2ng/ml) with SMAD inhibitor (SB431542, 0.5 μM). β-actin is used as loading control.

TGF-β is known to inhibit the proliferation of most target cells by SMAD-mediated signaling [34,35]. Cellular proliferative responses to TGF-β in mesenchymal cells have been described, although mechanisms are not well defined. We examined the effect of TGF-β1 on the proliferative responses of early passage non-transformed human lung fibroblasts (IMR-90). Our studies show that TGF-β1 (2 ng/ml) consistently inhibits the proliferation of IMR-90 fibroblasts grown in the presence of serum (10% FBS) as assessed by cell number/Coulter counter (Fig. 1A) and by BrdU incorporation at 48 h following TGF-β1 stimulation (Fig. 1B). Pre-treatment with the ALK5/SMAD inhibitor (SB431542; 0.5 μM) blocked the growth-inhibitory effect of TGF-β1 (Fig. 1A, B). Under the same conditions, TGF-β1 mediates the down-regulation of cyclin D1 in these cells, an effect that is also abrogated by ALK5/SMAD inhibition (Fig. 1C).

Inhibition of cell proliferation may be associated with, and may promote/facilitate, the induction of cellular differentiation responses [36]. We have previously shown that TGF-β1 is a potent inducer of myofibroblast differentiation in quiescent, serum-starved IMR-90 fibroblasts [27]. To determine if inhibition of serum-stimulated fibroblast proliferation by TGF-β1 is associated with the stimulation of myofibroblast differentiation, we assessed cellular expression of α-smooth muscle actin (α-SMA), a marker of myofibroblast differentiation. TGF-β1 induced time-dependent protein expression of α-SMA (Fig. 1D) and the formation of α-SMA-containing stress fibers (Fig. 1E). As with the observed effects of ALK5/SMAD blockade (with SB431542) on cyclin D1 and associated growth-inhibition induced by TGF-β1, the up-regulation of α-SMA was also inhibited by SB431542 (Fig. 1C; middle panel). Immunofluorescent staining demonstrated robust formation of α-SMA-containing stress fibers in TGF-β differentiated cells, an effect that inhibited in the presence of SB431542 (Fig. 1E); this inhibition was also observed in α-SMA protein expression by western blotting (Fig. 1F). Together, these results demonstrate that the “primary” effect of TGF-β1 on cultured non-transformed human lung fibroblasts is to induce a program of growth-arrest and cellular differentiation that require ALK5/SMAD signaling.

### Cav-1 gene and protein expression are down-regulated during the induction of myofibroblast differentiation by TGF-β1

**Figure 2:**
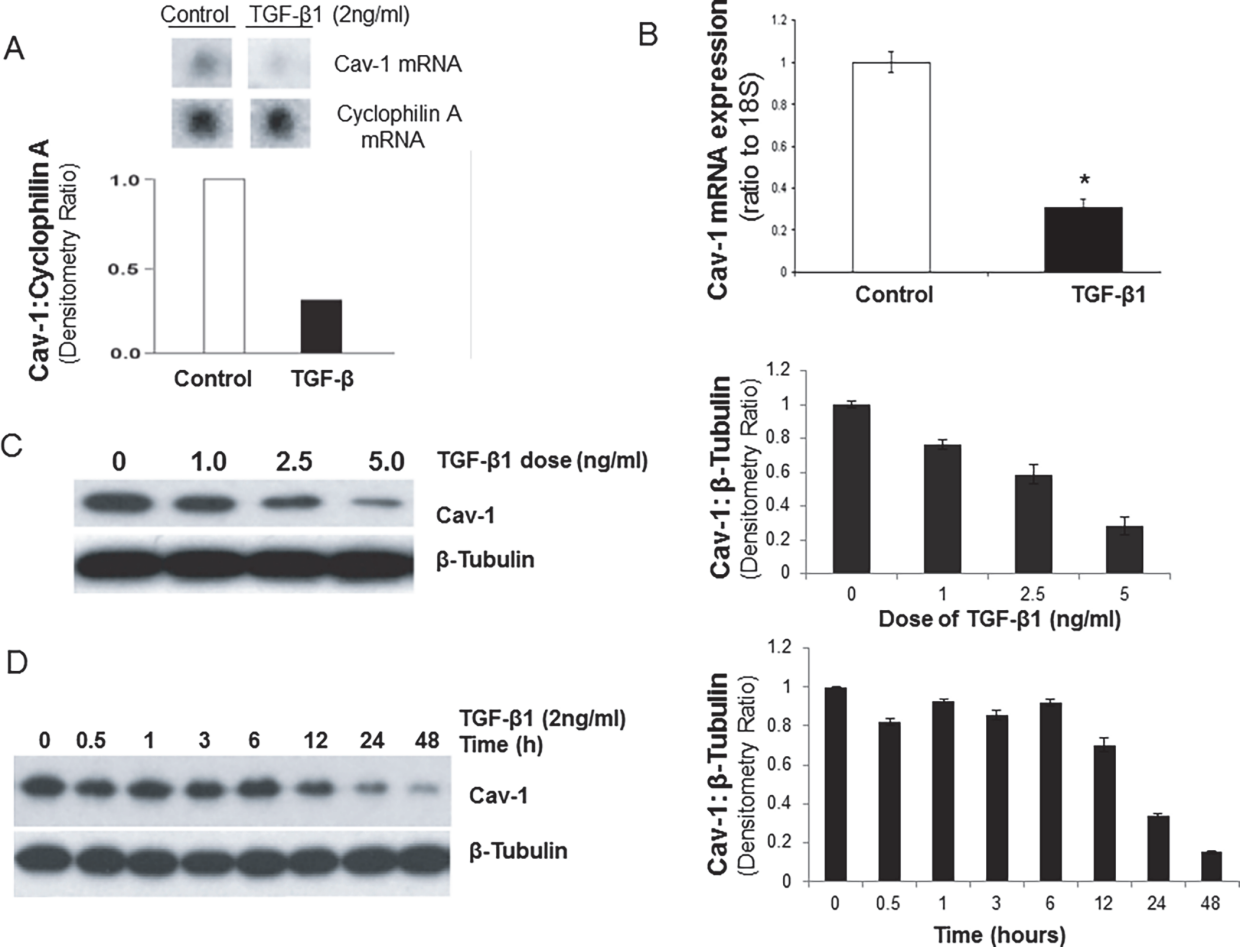
TGF-β1 induces dose- and time-dependent down-regulation of CAV-1 in human lung fibroblasts. ***A and B***, Cultured IMR-90 cells were treated with or without TGF-β1 (2 ng/ml) for 24 h and total RNA was isolated. A, cDNA Superarray analysis was performed and mRNA expression levels of Cav-1 and cyclophilin A are shown. Densitometric ratios of Cav-1:cyclophilin A are also shown. B, Histogram of real-time RT-PCR for Cav-1 expression after treated with TGF-β1 (2 ng/ml for 24 h) using triplicate samples from at least three individual experiments, normalized to 18S as mean±S.E.M. *p<0.05 compared to control. ***C***, IMR-90 cells were treated with TGF-β1 (0–5 ng/ml) for 24 h and cell lysates extracted. Cell lysates were subjected to SDS-PAGE and immunoblotted with an antibody against Cav-1; the blot was stripped and probed for β-tubulin. Densitometric ratios of Cav-1:β-tubulin are also shown on the right, as mean±S.E.M. **D**, IMR-90 cells were stimulated with TGF-β1 (2 ng/ml) for the indicated times. Cell lysates were subjected to SDS-PAGE and immunoblotted with an antibody against Cav-1; the blot was then stripped and probed for β-tubulin. Densitometric ratios of Cav-1:β-tubulin are shown on the right, as mean±S.E.M. Results are averages of at least three independent experiments.

TGF-β1 mediates anti-proliferative effects on target cells through the activation of its receptor serine-threonine kinase(s) complex [2]. Additionally, TGF-β1 may “prime” mesenchymal cells to proliferate in response to activation of classical receptor tyrosine kinases (RTKs) [10,12,37]. Down-regulation of Cav-1 may “prime” cells for proliferation in response to secondary extracellular mitogenic stimuli [38,39]. We examined the effect of TGF-β1 on the regulation of Cav-1 mRNA and protein expression in IMR-90 fibroblasts that simultaneously undergo myofibroblast differentiation in response to TGF-β1 stimulation. TGF-β1 (2 ng/ml) induced marked reduction in Cav-1 mRNA by 24 h in IMR-90 cells when assessed by cDNA Superarray analysis (Fig. 2A; Cav-1 and Cyclophilin A cDNA wells from the array are shown). This was confirmed with real-time RT-PCR (Fig. 2B). Dose-dependent effects of TGF-β1 (0, 1, 2.5 and 5 ng/ml) on the down-regulation of Cav-1 expression were confirmed by Western blot analysis (Fig. 2C). This effect of TGF-β1 (2 ng/ml) was time-dependent with a steady decline in Cav-1 protein expression up to 48 h when Cav-1 protein expression is reduced to ∼ 80% of baseline levels (Fig. 2D). Thus, TGF-β1 mediates a dose- and time-dependent down-regulation of Cav-1 expression in cells that are undergoing myofibroblast differentiation.

### TGF-β1-induced myofibroblast differentiation is SMAD-dependent, while Cav-1 down-regulation is p38 MAPK-dependent and SMAD-independent

**Figure 3:**
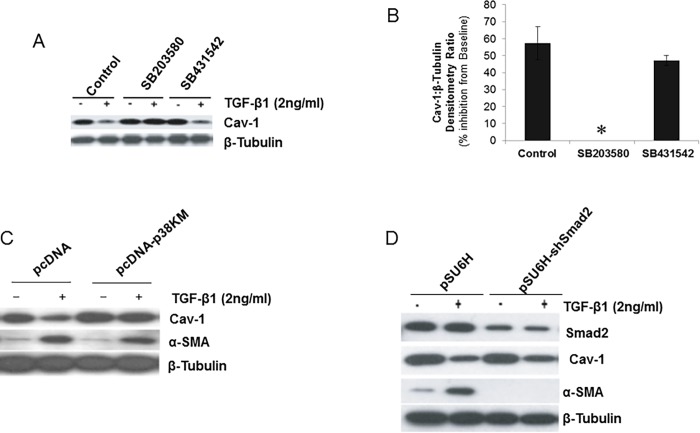
Down-regulation of Cav-1 by TGF-β1 is mediated by p38 MAPK-dependent and SMAD-independent mechanisms. ***A***, IMR-90 cells were treated with inhibitors of p38 MAPK (SB203580; 6 μM) or ALK5 (SB431542; 0.5 μM) for 30 min prior to treatment with or without TGF-β1 (2 ng/ml) for a period of 48 h. Cell lysates were extracted and Western immunoblotting performed with an antibody against Cav-1; the blot was then stripped and probed for β-tubulin. ***B***, Densitometric analyses of blots in (A) showed as % inhibition of baseline Cav-1 protein expression levels treated with TGF-β1. *indicates effect of SB203580 to completely block the inhibitory effect of TGF-β1 on Cav-1 expression. Results are averages of at least three independent experiments. Data are presented as mean±S.E.M. ***C***, IMR-90 cells stably transfected with a kinase-deficient p38 MAPK (pcDNA-p38KM) or control vector (pcDNA) were treated with or without TGF-β1 (2 ng/ml) for 24 h. Cell lysates were obtained and subjected to SDS-PAGE and immunoblotted for Cav-1 and α-smooth muscle actin (α-SMA); blots were stripped and probed for β-tubulin. ***D***, IMR-90 cells stably expressing SMAD2 *sh*RNA (pSU6H-*sh*SMAD2) or control vector (pSU6H) were treated with/without TGF-β1 (2 ng/ml) for 24 h. Cell lysates were immunoblotted for SMAD2, Cav-1 and α-SMA. The blots were stripped and probed for β-tubulin.

TGF-β receptor(s) signaling activates both SMAD-dependent and –independent pathways [2,35]. We have previously demonstrated rapid activation of p38 MAPK by TGF-β1 in human lung fibroblasts/mesenchymal cells, including IMR-90 fibroblasts [28]. Here, we determined the potential role(s) of early TGF-β receptor(s)-activated SMAD-dependent and –independent p38 MAPK activation in the down-regulation of Cav-1 expression by TGF-β1. First, we assessed the effects of pharmacologic inhibitors of p38 MAPK (SB203580; 6 μM) and ALK5/SMAD (SB431542; 0.5 μM) on TGF-β1-mediated down-regulation of Cav-1 expression. Pharmacologic inhibition of p38 MAPK blocked TGF-β1-induced down-regulation of Cav-1 protein expression, while inhibition of ALK5/SMAD signaling did not (Fig. 3A, B). Inhibitors of ERK-1/2 MAPK (with the MEK-1 inhibitor, PD98059, 20 μM), c-Jun N-terminal kinase (JNK-II, 0.1 μM) and Src family protein tyrosine kinases (PP2, 10 μM and SU6656, 2.7 μM) had no effect (data not shown). To confirm the role of p38 MAPK in TGF-β1-induced down-regulation of Cav-1 expression, we employed IMR-90 cells over-expressing a kinase-mutant p38 MAPK that is phosphorylated on Thr180/182, but is not catalytically active as previously described [28]. In cells expressing mutant p38 MAPK (p38-KM), TGF-β1 failed to down-regulate Cav-1 protein expression, while this effect was well-preserved in control vector-transfected cells (pcDNA) (Fig. 3C). In contrast to effects on Cav-1 expression, blockade of p38 MAPK signaling does *not* alter the ability of TGF-β1 to induce α-SMA expression (Fig. 3C).

To then define the role of SMAD signaling in these TGF-β1 effects, cells were stably transfected with a plasmid encoding *sh*RNA against SMAD2 (pSU6H-*sh*SMAD2). Knock-down of SMAD2 in IMR-90 fibroblasts completely blocks the up-regulation of α-SMA, but has no effect on Cav-1 down-regulation by TGF-β1 (Fig. 3D). Similar results were noted in SMAD3 knock-down cells (data not shown). These results demonstrate that myofibroblast differentiation and Cav-1 down-regulation are independently regulated via the SMAD and p38 MAPK signaling pathways, respectively.

### Overexpression of Cav-1 blocks the “priming” effect of TGF-β on proliferation and resistance to apoptosis of myofibroblasts

**Figure 4:**
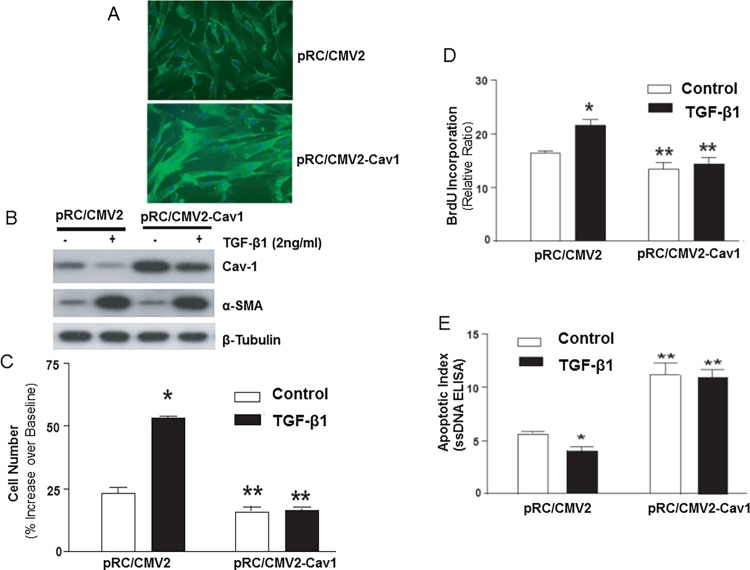
Over-expression of Cav-1 inhibits proliferation of human lung fibroblasts/myofibroblasts and abrogates the anti-apoptotic effects of TGF-β1. ***A***, Human lung fibroblasts (IMR-90) were stably transfected with a plasmid encoding Cav-1 (pRC/CMV2-Cav1) or with empty vector (pRC/CMV2). Localization of Cav-1 protein was then analyzed by immunofluoresence staining with a rabbit polyclonal antibody to Cav-1. ***B***, Stably-transfected cells described in (A) were treated with/without TGF-β1 (2 ng/ml) for 24 h. Cell lysates were obtained and subjected to immunoblotting for Cav-1, α-smooth muscle actin (α-SMA), and β-tubulin. ***C***, Stably-transfected cells described in (A) were serum-deprived for 24 h and then treated with/without TGF-β1 (2 ng/ml) for 48 h followed by stimulation with 10% fetal bovine serum for 24 h. Cell numbers were assessed both prior to and after serum stimulation with an automated Coulter counter (n = 6 per group) as mean±S.E.M. *indicates *p* < 0.05 vs. control pRC/CMV2 “fibroblasts”. **indicates *p* < 0.05 vs. pRC/CMV2 “myofibroblasts” (TGF-β1 pre-treated). Similar results were obtained from 3 independent experiments. ***D***, Cells in (C) were labeled with BrdU during the 24 h of serum stimulation (n = 6 per group). BrdU assays were performed as described in “Methods”. *indicates *p* < 0.05 vs. control pRC/CMV2 “fibroblasts”. **indicates *p* < 0.05 vs. pRC/CMV2 “myofibroblasts” (TGF-β1 pre-treated). Similar results were obtained from 3 independent experiments. ***E***, Quiescent stably transfected IMR-90 cells described in (A) were treated with/without TGF-β1 (2 ng/ml) for 5 days in serum-free medium. Apoptosis assays using an ELISA for *ss*DNA (n = 6 for each group) were performed as described in “Methods”. *indicates *p* < 0.05 vs. control pRC/CMV2 “fibroblasts”. **indicates *p* < 0.01 vs. pRC/CMV2 “myofibroblasts” (TGF-β1 pre-treated). Similar results were obtained from 3 independent experiments.

Cav-1 down-regulation may “prime” cells for enhanced proliferation in response to exogenous mitogens and the cells acquire an apoptosis-resistant phenotype. We have previously shown that TGF-β1-treated IMR-90 cells proliferate more robustly in response to exogenous mitogens [12] and that they are more resistant to serum deprivation-induced apoptosis [28]. Whether these effects may, in part, be mediated by the down-regulation of Cav-1 is not known. To examine the role(s) of Cav-1 down-regulation in these physiological responses to TGF-β1, Cav-1 protein was stably over-expressed in IMR-90 fibroblasts. In these cells, Cav-1 protein expression is primarily localized to the plasma membrane by immunofluorescence staining (Fig. 4A). Cav-1 over-expressing cells (pRC/CMV2-Cav1) maintained their ability to up-regulate α-SMA in response to TGF-β1 (Fig. 4B), suggesting that Cav-1 over-expression does not interfere with TGF-β1-induced myofibroblast differentiation. We noted down-regulation of Cav-1 protein expression by TGF-β1 even in pRC/CMV2-Cav1 cells; however, Cav-1 protein levels do not decline below baseline/control levels in cells expressing the empty vector (pRC/CMV2) (Fig. 4B).

We then determined if over-expression of Cav-1 can mitigate the hyper-proliferative response and the apoptosis-resistant phenotype of myofibroblasts. Cav-1 over-expressing cells (pRC/CMV2-Cav1) and control vector-expressing cells (pRC/CMV2) were treated with (or without) TGF-β1 (2 ng/ml) in the absence of serum for 48 h to induce (or not induce) myofibroblast differentiation; all cells were then stimulated with serum (10% FBS) for 24 h. In pRC/CMV2 (control) cells, serum stimulation induced proliferative responses in both fibroblasts and myofibroblasts as assessed by cell number/Coulter counting and BrdU incorporation; this proliferative response was significantly greater in TGF-β1-differentiated myofibroblasts (Fig. 4C, D). Over-expression of Cav-1 abrogated the accentuated proliferative response in myofibroblasts (Fig. 4C, D), suggesting that down-regulation of Cav-1 represents an important mechanism for sensing and regulating extracellular serum-stimulated mitogenic signals in myofibroblasts. Cav-1 over-expression alone also suppressed, although to a lesser degree, the proliferative response to serum in undifferentiated fibroblasts (Fig. 4C, D).

We have previously shown that TGF-β1-differentiated myofibroblasts are resistant to serum deprivation-induced apoptosis [28]. This apoptosis-resistant myofibroblast phenotype was maintained in cells stably transfected with a control vector (pRC/CMV2) when subjected to serum-deprivation for 5 days (Fig. 4E). Cav-1 over-expressing cells (pRC/CMV2-Cav1), however, demonstrated significantly higher rates of apoptosis in fibroblasts and myofibroblasts (Fig. 4E). This loss of TGF-β1-induced protection from apoptosis in Cav-1-over-expressing cells suggests that down-regulation of Cav-1 is a critical regulatory step for the acquisition of an apoptosis-resistant phenotype in myofibroblasts.

### Stable shRNA knockdown of Cav-1 promotes myofibroblast proliferation and survival

**Figure 5:**
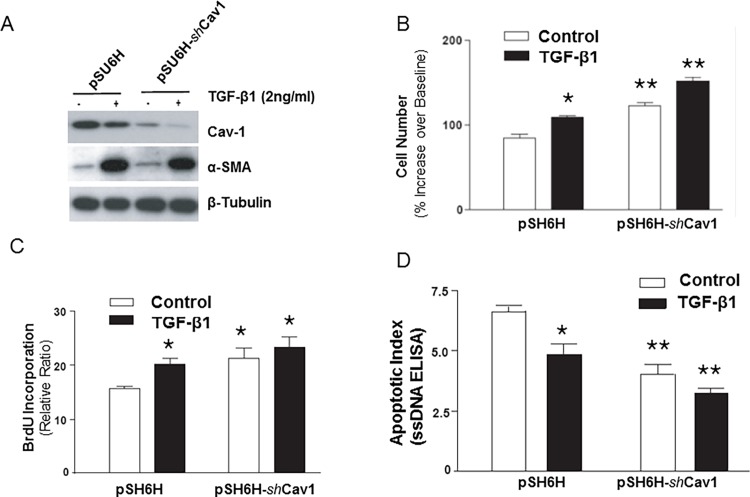
*sh*RNA knock-down of Cav-1 enhances fibroblast/myofibroblast proliferation and protects against serum deprivation-induced apoptosis. ***A***, IMR-90 cells stably transfected with a plasmid encoding *sh*RNA targeted against Cav-1 (pSU6H-*sh*Cav1) or with control plasmid (pSU6H) were treated with/without TGF-β1 (2 ng/ml) for 24 h. Cell lysates were obtained and Western blots for Cav-1, α-smooth muscle actin (α-SMA) and β-tubulin performed. ***B***, Stably-transfected cells described in (A) were serum-deprived for 24 h and treated with/without TGF-β1 (2 ng/ml) for 48 h followed by stimulation with 10% FBS for 24 h. Cell counts were assessed both prior to and after serum stimulation with an automated Coulter counter (n = 6 per group) shown as mean±S.E.M. *indicates *p* < 0.05 vs. control pSU6H “fibroblasts”. **indicates *p* < 0.05 vs. pSU6H “myofibroblasts” (TGF-β1 pre-treated). Similar results were obtained from 3 independent experiments. ***C***, Stably transfected cells described in (A) were serum-deprived for 24 h and treated with/without TGF-β1 (2 ng/ml) for 48 h followed by BrdU labeling for 24 h in the presence of 10% FBS (n = 6 per group) shown as mean±S.E.M. *indicates *p* < 0.05 vs. control pSU6H “fibroblasts”. Similar results were obtained from 3 independent experiments. ***D***, Quiescent stably-transfected cells described in (***A***) were treated with/without TGF-β1 (2 ng/ml) for 5 days and apoptotic assay for *ss*DNA performed as described in “Methods” (n = 6 per group) shown as mean±S.E.M. *indicates *p* < 0.05 vs. control pSU6H “fibroblasts”. **indicates *p* < 0.05 vs. pSU6H “myofibroblasts” (TGF-β1 pre-treated). Similar results were obtained from 3 independent experiments.

We next assessed the effects of Cav-1 knockdown (by stable expression of *sh*RNA to Cav-1) on fibroblast/myofibroblast proliferation and resistance to apoptosis. Knockdown of Cav-1 at the protein level was confirmed by Western immunoblotting (Fig. 5A, top panel). TGF-β1 induced myofibroblast differentiation, as determined by α-SMA expression, in *both* control cells (pSU6H; transfected with empty vector) and Cav-1 knock-down cells (pSU6H-*sh*Cav1) (Fig. 5A). Proliferative responses to serum stimulation (10% FBS for 24 h) were significantly augmented in Cav-1 knock-down cells, both in control fibroblasts and myofibroblasts (cell pre-treated with TGF-β1 for 48 h) when assessed by cell numbers (Fig. 5B) and BrdU incorporation (Fig. 5C). Serum deprivation-induced apoptosis of both fibroblasts and myofibroblasts was suppressed in Cav-1 knock-down cells (Fig. 5D). Interestingly, TGF-β1 confers additional protection from apoptosis even in cells deficient in Cav-1 (Fig. 5D), which may be related, in part, to further a decrease in Cav-1 expression observed in these cells (Fig. 5A). These results further support the notion that down-regulation of Cav-1 represents an important regulatory mechanism for enhanced proliferative and apoptosis-resistant phenotypes of mesenchymal cells.

### Activation of p38 MAPK, but not SMAD signaling, mediates augmented proliferative responses to serum in myofibroblasts induced by TGF-β

**Figure 6:**
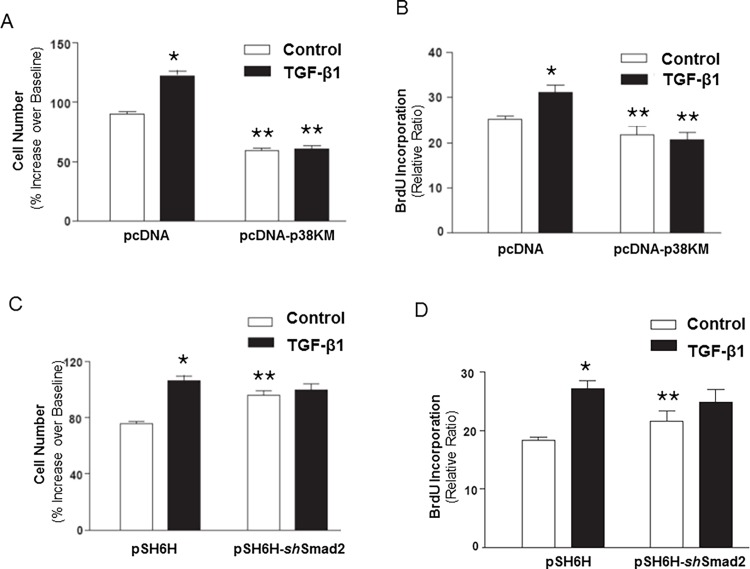
Blocking p38 MAPK inhibits myofibroblast proliferation. ***A***, IMR-90 cells stably expressing a p38 MAPK dominant negative (pcDNA-p38KM) and cells stably transfected with an empty vector (pcDNA) were serum-starved for 24 h and treated with/without TGF- β1 (2 ng/ml) for 48 h followed by stimulation with 10% fetal bovine serum for 24 h (n = 6 per group), data shown as mean±S.E.M. *indicates *p* < 0.05 vs. control pcDNA “fibroblasts”. **indicates *p* < 0.01 vs. control pcDNA “myofibroblasts” (TGF-β1 pre-treated). Similar results were obtained from 3 independent experiments. ***B***, Cells described in (A) were grown in 96-well plates and serum-starved for 24 h and treated with/without TGF- β1 (2 ng/ml) for 48 h followed by BrdU labeling for 24 h in the presence of 10% fetal bovine serum (n = 6 per group), data shown as mean±S.E.M. *indicates *p* < 0.05 vs. control pcDNA “fibroblasts”. **indicates *p* < 0.01 vs. control pcDNA “myofibroblasts” (TGF-β1 pre-treated). Similar results were obtained from 3 independent experiments. ***C***, IMR-90 cells stably expressing SMAD2 *sh*RNA and cells stably transfected with an empty vector (pSU6H) were serum-starved for 24 h and treated with/without TGF- β1 (2 ng/ml) for 48 h followed by stimulation with 10% fetal bovine serum for 24 h (n = 6 per group) data shown as mean±S.E.M. *indicates *p* < 0.05 vs. control pSU6H “fibroblasts” (no TGF-β1 pre-treatment). **indicates *p* < 0.05 vs. control pcDNA “myofibroblasts” (TGF-β1 pre-treated). ***D***, Cells described in (C) were grown in 96-well plates and serum-starved for 24 h and treated with/without TGF- β1 (2 ng/ml) for 48 h followed by BrdU labeling for 24 h in the presence of 10% fetal bovine serum (n = 6 per group) data shown as mean±S.E.M. *indicates *p* < 0.05 vs. control pSU6H “fibroblasts” (no TGF-β1 pre-treatment). **indicates *p* < 0.05 vs. control pcDNA “myofibroblasts” (TGF-β1 pre-treated).

The TGF-β/SMAD pathway is well recognized to mediate growth-inhibitory and tumor-suppressive effects [2]. In some contexts, growth-promoting effects may be observed [8–12]. Our data indicate that early activation of the p38 MAPK pathway by TGF-β1 mediates the down-regulation of Cav-1 expression during the process of fibroblast to myofibroblast differentiation. Based on our model, blockade of the upstream activation of p38 MAPK should inhibit the proliferative responses of myofibroblasts to mitogenic stimulation. We first treated IMR-90 fibroblasts stably expressing a control vector (pcDNA) or kinase-deficient p38 MAPK (pcDNA-p38KM) with or without TGF-β1 (2 ng/ml × 48 h) to differentiate them into myofibroblasts or maintain them as undifferentiated fibroblasts. We have already shown that blockade of the p38 MAPK pathway does not alter myofibroblast differentiation, but is critical for the down-regulation of Cav-1 in TGF-β1-stimulated IMR-90 cells (Fig. 3). Following differentiation into myofibroblasts, cells were stimulated with/without serum (10% FBS) for 24 h to assess proliferative responses. Both fibroblasts and myofibroblasts responded robustly to serum stimulation; however, the proliferative rates were consistently greater in myofibroblasts compared to fibroblasts (Fig. 6A, B: pcDNA control cell lines; and Fig. 6C, D: pSU6H control cell lines). This “growth advantage” was completely lost in p38KM cells, supporting a role for p38 MAPK in this proliferative response of myofibroblasts (Fig. 6A, B). A statistically significant decrease in proliferative capacity was also noted in fibroblasts (non-TGF-β1-treated) expressing p38KM, suggesting that constitutive p38 MAPK (in the absence of TGF-β1 signaling) may also regulate this growth-promoting pathway. In support of this concept, baseline levels of Cav-1 expression were noted to be elevated in p38KM cells (Fig. 3C, compare 3^rd^ and 4^th^ lanes to 2^nd^ lane).

To examine the role of SMAD in proliferative signaling of mesenchymal cells, we generated IMR-90 cells stably expressing shRNA for SMAD2 (pSU6H-*sh*SMAD2) and the control vector (pSU6H). In contrast to the observed effects of blockade of p38 MAPK signaling, knock-down of SMAD2 did not significantly alter the proliferative rate of myofibroblasts by both cell counting and BrdU incorporation (Fig. 6C, D), suggesting that p38 MAPK and not the SMAD2 signaling is required for the accentuated proliferation of myofibroblasts. In fact, SMAD2 knock-down cells demonstrated slightly *higher* baseline rates of cell proliferation (Fig. 6C, D), consistent with the role of the TGF-β/SMAD pathway in cell cycle arrest and tumor suppression. We observed no further augmentation of cell proliferation in TGF-β1 pre-treated cells in the context of stable SMAD2-deficieny (Fig. 6C, D), suggesting that the growth advantage acquired as a result of the blockade of SMAD signaling is not further enhanced by down-regulation of Cav-1.

## DISCUSSION

**Figure 7:**
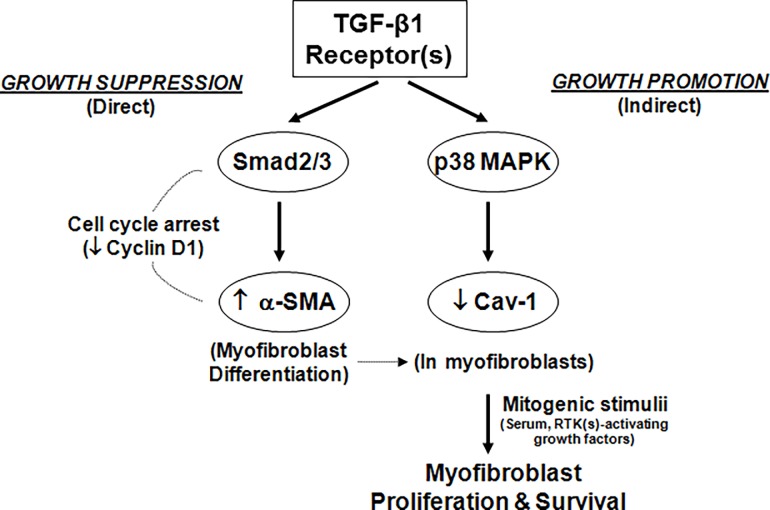
Schematic representation of TGF-β1-activated signaling pathways mediating mesenchymal cell growth -suppressive and -promoting effects. TGF-β1 activates the cell surface TGF-β receptor(s) complex that leads to rapid activation of the canonical SMAD pathway as well as the SMAD-*in*dependent p38 MAPK pathway. Activation of the SMAD pathway is required for the induction of a cellular program of growth-arrest and myofibroblast differentiation. In contrast, activation of the p38 MAPK pathway, independently of SMAD2/3, is required the down-regulation of Cav-1 by TGF-β1. Down-regulation of Cav-1 by TGF-β1 “primes” differentiated myofibroblasts for enhanced proliferative responses to mitogens and resistance to apoptosis. These divergent TGF-β signaling pathways may explain, in part, the contextual effects of TGF-β1 as both a growth-inhibitor and –promoter on the same target (mesenchymal) cells.

The complex biology of TGF-β is exemplified by its well-recognized, yet contrasting, physiological roles in both tumor-suppression and tumor-promotion [40]. A similar, and somewhat perplexing, observation is that TGF-β1, signaling via the same TGF-β receptor(s) complex, mediates apparent growth-promoting effects in mesenchymal cells while mediating primarily growth-inhibitory effects on most other cell types. While the canonical SMAD proteins, interacting with other transcriptional complexes within the nucleus, may explain some these contextual actions [2], SMAD-independent pathways activated at the plasma membrane and in the cytosol are also likely to be important. We report that, while the primary effect of TGF-β1 is to mediate growth-inhibition and myofibroblast differentiation via a SMAD-dependent pathway in non-transformed human lung fibroblasts, secondary effects on myofibroblast proliferation and survival are mediated by an alternate SMAD-*in*dependent, and p38 MAPK-dependent, pathway that mediates the concomitant down-regulation of Cav-1 in differentiated myofibroblasts (Fig. 7). This provides a novel mechanism by which TGF-β1 mediates “growth-suppressive” and “growth-promoting” effects on the *same* target cell, under *different* contexts.

There is growing recognition that plasma membrane caveolae microdomains regulate diverse cellular functions [17]. Insights into the in-vivo physiological functions of caveolae have been gained from targeted disruption in mice of Cav-1, the main protein component of caveolae [26]. A prominent phenotype of Cav-1-deficient mice are the pulmonary abnormalities characterized by thickened alveolar septae and fibrotic changes involving the interstitium of the lung [26]; yet current understanding of Cav-1 function in fibroblasts/myofibroblasts, key effector cells of the fibrotic response, is limited. Although Cav-1 plays an important role in lung diseases [41], regulatory mechanisms that control Cav-1 gene/protein expression by growth factors have not been well defined. TGF-β1 is a central regulator of tissue fibrosis in most mammalian organ systems, including the lung [16,42,43]. In this study, we show that TGF-β1 down-regulates Cav-1 expression in association with myofibroblast differentiation in non-transformed human lung fibroblasts. Few growth factor ligands have been shown to directly regulate gene/protein expression of Cav-1 in mammalian cells. Our study is similar to another report that TGF-β1 mediates the down-regulation of Cav-1 in human lung fibroblasts [44,45] and studies showed the decreased Cav-1 in IPF fibroblasts [44,45]. Activation of the Ras-MAPK pathway and protein kinase A has been shown to suppress Cav-1 gene expression [46]; thus, mitogenic factors in serum may be capable of inducing this effect. Chronic exposure to epidermal growth factor (EGF) has been recently reported to decrease Cav-1 expression in epithelial cancer cells that leads to the loss of E-cadherin and an epithelial-mesenchymal transition [47]. Interestingly, TGF-β1 is a major regulator and inducer, of EMT [48,49].

TGF-β family members signal via heteromeric transmembrane complexes of type I and type II serine-threonine receptor kinases. The well-known direct effectors of TGF-β receptor(s) signaling are SMAD proteins that, when activated, function as transcriptional regulators [2]. More recently, early post-receptor signaling via SMAD-independent pathways have been increasingly recognized [35]. SMAD-independent activation of p38 MAPK by TGF-β1 has been previously reported in epithelial cells [50,51] and mesenchymal cells [28]. Our current studies clearly implicate p38 MAPK activation in the down-regulation of Cav-1 by TGF-β1 in human lung fibroblasts. Previous studies from our laboratory have also implicated early activation of the p38 MAPK pathway in the autocrine induction of growth factors that “protect” mesenchymal cells from serum deprivation-induced apoptosis [28]. Given the new findings in this study showing p38 MAPK-dependent down-regulation of Cav-1, we speculate that multiple “anti-apoptotic/pro-survival” mechanisms that coordinately interact with each other are activated by this SMAD-*in*dependent pathway. Our studies also support the concept that myofibroblast differentiation is regulated by a SMAD-dependent, yet p38 MAPK-*in*dependent, mechanism. Signaling via SMAD proteins have been previously implicated in myofibroblast differentiation induced by TGF-β1 [52,53]. Taken together, these data provide evidence for divergent signal transduction pathways involving a SMAD-dependent pathway that mediates growth-suppression and myofibroblast differentiation, on the one hand, and a p38 MAPK pathway that mediates growth-promoting and anti-apoptotic effects by autocrine production of growth factors in concert with the down-regulation of Cav-1, on the other (Fig. 7).

In comparison to endothelial and epithelial cells, studies of Cav-1 and caveolae function in mesenchymal cells/fibroblasts are limited. Our studies clearly demonstrate that down-regulation of Cav-1 augments the proliferative capacity of fibroblasts, while reducing rates of serum deprivation-induced apoptosis; in contrast, over-expression of Cav-1 lowers baseline proliferative capacity and increases apoptotic rates. Importantly, over-expression of Cav-1 abrogates, in large part, the enhanced proliferative capacity of *myo*fibroblasts and the anti-apoptotic phenotype of *myo*fibroblasts. The implications of these findings are that the selective blockade of p38 MAPK signaling may effectively serve to “suppress” myofibroblast activity—specifically, the proliferation and survival of myofibroblasts—without interfering with myofibroblast differentiation *per se*. Moreover, preservation of SMAD signaling is likely to be critical for important homeostatic functions of TGF-β on other cell types: “tumor-suppression” in epithelial cells and “immune-suppression” in immune cells [42].

In the current study, we have not explored the mechanisms by which p38 MAPK down-regulates Cav-1. There are some plausible mechanisms; for example, previous studies demonstrate that GATA-6 binds to the Cav-1 promoter to down-regulate its transcription/gene expression [54], and that p38 MAPK can directly regulate the levels of GATA-6 via miR17-92 [55]. Other possible mechanisms may involve p38 MAPK-activated epigenetic regulation of Cav-1 expression [56], which requires further investigation.

In summary, our study proposes a novel mechanism for the proliferation and survival of myofibroblasts that involves coordinate, and independently regulated, suppression of Cav-1 by TGF-β1. Cav-1 is typically up-regulated in terminally differentiated cells [39]; the down-regulation of Cav-1 in “differentiated” myofibroblasts may explain the unusual capacity of these cells to continue to proliferate and, in some contexts, to escape apoptosis [57]. Such mechanisms are likely to be important in chronic injury and repair processes characterized by the persistence of myofibroblasts in injured/fibrotic tissues that eventually progresses to end-organ failure. The potential roles for TGF-β1 regulation of Cav-1 in tumor biology and fibrosis require further study.
